# Two Sides of the Same Coin in Female Borderline Personality Disorder: Self-Reported Guilt and Shame and Their Neurofunctional Correlates

**DOI:** 10.3390/brainsci14060549

**Published:** 2024-05-27

**Authors:** Hella Parpart, Jakob Blass, Thomas Meindl, Janusch Blautzik, Petra Michl, Thomas Beblo, Rolf Engel, Maximilian Reiser, Peter Falkai, Hans-Juergen Moeller, Martin Driessen, Kristina Hennig-Fast

**Affiliations:** 1 Department of Psychiatry and Psychotherapy, LMU Hospital, 80336 Munich, Germany; 2 Department of Psychiatry and Psychotherapy, Universitätsklinikum OWL, 33617 Bielefeld, Germany; 3 Institute of Clinical Radiology, LMU Hospital, 80336 Munich, Germany

**Keywords:** social emotion, shame, guilt, borderline personality disorder, fMRI

## Abstract

Objective: Patients with borderline personality disorder (BPD) report to be especially prone to social emotions like shame and guilt. At the same time, these emotions seem to play an important role in BPD pathology. The present study aimed to deepen the knowledge about the processes behind shame and guilt in patients with BPD. Methods: Twenty patients with BPD and twenty healthy controls (HCs) took part in an experiment that induced shame and guilt by imagining scenarios during scanning using functional brain imaging. Participants also filled out self-report questionnaires and took part in diagnostic interviews. Results: BPD patients reported more proneness to guilt but not to shame than the HCs. There was no difference in the self-reported intensity rating of experimentally induced emotions between the groups. Between-group contrast of neural signals in the shame condition revealed a stronger activation of cingulate and fusiform gyrus for the BPD patients compared to the controls, and a more pronounced activation in the lingual gyrus and cuneus for the HCs. In the guilt condition, activation in the caudate nucleus, the fusiform gyrus, and the posterior cingulate cortex was stronger in BPD patients, while HC showed stronger activations in cuneus, lingual gyrus, and fronto-temporal regions. Conclusions: Differences in the neuro-functional processes between BPD patients and HC were found, even though the two groups did not differ in their self-report of subjective proneness to guilt and emotional intensity of shame and guilt during the experiment. While the HCs may be engaged more by the emotional scenarios themselves, the BPD patients may be more occupied with cognitive regulatory and self-referential processing.

## 1. Introduction

Borderline personality disorder (BPD) is most often described by symptoms of interpersonal problems, a sense of unstable identity, and emotional dysregulation [1]. Among other emotions, the regulation of shame and guilt in particular is pathological in patients with BPD, resulting in heightened self-reported levels of both emotions compared to subjects with other psychological disorders and healthy controls (HC) [2,3] and seems to play an important role in BPD psychopathology [4]. Both emotions are accompanied by negative affective states as a result of a social transgression (e.g., the violation of social norms). Guilt is described as the feeling of being responsible for a distinct wrong or misfortune. It is a result of internal attribution of unstable, distinct behaviors, and can motivate prosociality, other-oriented empathy, and reparation [5]. Opposed to this, shame is described as the feeling of being wrong or bad as a person and results from internal attribution of stable, global self-evaluations. Shame promotes self-defense, denial, and avoidance [5]. Thus, guilt is thought to be an adaptive and shame to be a maladaptive social emotion for psychological well-being. Schoenleber and Berenbaum [6] proposed three groups of maladaptive regulation attempts closely related to shame that lead to the maintenance of BPD symptoms: prevention (e.g., dependence, fantasy), escape (e.g., social withdrawal, misdirection), aggression (against one-self and others). Fittingly, some evidence hints that shame (or the proneness for it) relates to maladaptive outcomes such as lower self-esteem, quality of life, and anger/hostility [7,8], internalizing and externalizing problems [9], as well as non-suicidal self-injury and suicidal ideation [10,11,12]. Increased reported guilt(-proneness) might protect against adverse outcomes, such as anger and aggression, in the context of BPD [13]. In a cross-sectional study, Cameron and colleagues [14] found that more shame-proneness but less guilt-proneness was associated with poorer psychosocial functioning. Aside from BPD, the role of shame and guilt for symptoms of depression [15], anxiety [16], social anxiety [17], obsessive compulsive disorder [18], PTSD [19], eating disorder [20], substance abuse [21], dissociation [22], and impulsivity [9] has been reported in numerous studies. Winter and colleagues [23] discuss that elevated proneness for aversive social emotions in BPD might be due unstable and negative self-concept (e.g., reported low self-esteem) and possibly altered self-related motives, which might lead to increased reactivity to self-relevant cues. In a recent experiment, Biermann et al. [24] showed that the focus on one-self vs. others seems to play a key role in explaining the altered social emotional reaction of BPD patients compared to HCs, which seems to be related to self-critical and self-harming processing. As shame and guilt are social emotions, they play a vital role in regulating social behavior and are influenced by one’s history of social learning. Fittingly, interpersonal trauma (or social traumatization), which is frequent in BPD [25], is thought to play an important role for the pathological manifestations of shame and guilt in psychological disorders [26]. Accordingly, there is a considerable body of evidence linking reports of heightened shame and guilt to interpersonal trauma and symptoms of trauma-associated disorders [27,28] as BPD [8,13]. Following the assumption that social emotions function as a compass in social situations [23], Dorahy [29] found shame (but not guilt) to be predictive of interpersonal problems. Conversely, interactional problems might contribute to the maintenance of dysfunctional emotional regulation [6]. Considering this, social emotions like shame and guilt could be seen as general factors that work through various mechanisms in psychopathology, and especially in BPD. A deeper understanding of the mechanisms of self-conscious emotions seems crucial to improving treatment of trauma-associated disorders like BPD.

### 1.1. Neural Representations of Shame and Guilt in General

A growing body of literature describes the neural correlates of shame and guilt in healthy subjects [30], derived from studies using functional magnetic resonance imaging (fMRI). Systematic reviews and meta-analyses found both shared and unique neural representations of shame and guilt. Most of the evidence supports an involvement of the anterior insula (AI; relevant for general emotional experience, inner awareness, and excitement) and the dorsal anterior cingulate cortex (dACC; social pain, negative emotional states, and their processing) in both emotions [31,32]. However, guilt is specifically associated with activation in the temporoparietal junction (TPJ; social cognitive processes), while shame corresponds to activation in the dorsolateral prefrontal cortex (dlPFC; cognitive and emotional regulatory processes), the cingulate cortex (processing of self-referential mental states) and the sensorimotor or premotor cortex [31,32].

### 1.2. Neural Representations of Shame and Guilt in BPD

Some studies found evidence for altered processing of social emotions in psychiatric patients compared to HCs [18,33]. Additionally, there is a growing body of evidence for the neural alterations associated with emotional dysregulation [34,35] and disturbed processing of social information [36] in BPD patients compared to HCs. For example, Orth and colleagues [37] conducted an experiment on the role of self-relevance. Classifying social information as relevant to oneself is a core process with regard to social emotions. In the experiment, BPD patients and HCs were compared with regard to sentences that were more or less relevant for themselves. The authors reported a higher connectivity between cortical midline structures and the basal ganglia–thalamus complex in BPD patients during processing of self-relevant information. Those regions are thought to be involved in salience detection and reward evaluation. Even recently, Göttlich et al. [38] conducted the first study to compare the neural processing of the social emotions shame and guilt between BPD patients and HCs. The paradigm used to induce shame and guilt consisted of three short sentences for each condition written in a third-person perspective presented repeatedly for 9 s. The authors found increased amygdala activity as well as decreased habituation of the amygdala activity in response to shame- and guilt-related stimuli in BPD patients compared to HCs. However, the differences between patients and HCs regarding the fMRI findings were rather small and shame and guilt could not be discriminated through neural signals. The authors attributed this in part to the comparatively few emotion induction sentences and the small sample size.

### 1.3. The Present Study

The present study aimed to first replicate and second to enhance the findings on shame and guilt and their neural processing in patients with BPD. To this end, we used an experimental paradigm [18] similar to the one by Göttlich et al. [38]. Specifically, we used more sentences to induce shame and guilt. Compared to the study of Göttlich et al. [38], the sentences focused more broadly on different aspects of both emotions. Furthermore, sentences were presented for a longer time period during the experimental procedure to consider the temporal dynamics within the limbic system [39]. We hypothesized BPD patients to report higher levels of shame and guilt than HCs in general (Hypothesis 1) and during the experimental stimulation (Hypothesis 2) in particular. Furthermore, we expected to find distinct clusters of neural activation for shame and guilt in the AI and the cingulate cortex. Beyond that, we expected clusters of activation to emerge in the dlPFC, the sensori- and premotor cortex for shame, and clusters in the TPJ to emerge during guilt (Hypothesis 3). We expected this activation to be more pronounced in BPD patients compared to HCs (Hypothesis 4).

## 2. Methods

The study design was approved by the Ethics Committee of the Medical Faculty of the Ludwig-Maximilians University in Munich (LMU; N° 301-12). The study was conducted between February and August 2014. The study participants with BPD were recruited on inpatient wards, in the day clinic and in the outpatient clinic of the Department of Psychiatry and Psychotherapy of LMU. The study was explained in detail to ward physicians and nursing staff. In addition, training outpatient clinics at various psychotherapy institutes were contacted and asked to participate. Flyers about the study were produced and distributed in all the aforementioned places. HCs were selected to ensure the best possible comparability in terms of educational level, intelligence quotient (IQ), and age with the patients suitable for the study. The HCs were recruited by personal approach and the study was also presented in two lectures of the bachelor program in psychology at the LMU. After initial contact and screening, participants were invited to the Department of Clinical Radiology of LMU. They received a detailed explanation of and written information about the study as well as risks and side effects of the MRI procedure and gave their informed consent. Prior to the scanning procedure, demographic information was assessed and clinical interviews were conducted. Participants then performed the experimental tasks in the MRI scanner for about 50 min. After the scanning procedure, participants filled out a series of self-report questionnaires. All participants received an allowance of 25 Euro for their expenses. All interviews and experimental assessments were conducted by trained psychologists with a diploma or a doctorate in psychology.

### 2.1. Sample

Forty female, right-handed participants were included in the study. Participants needed to be between 18 and 50 years old, so that they were of legal age to be able to consent to the study, but also to be able to rule out age-related brain changes. All participants needed to be right-handed to avoid modulating effects on hemispheric lateralization. Also, they had to have more than 85 IQ points, as measured with a language-based intelligence test [40], to ensure their capability to consent to the experimental procedure. Subjects were excluded from the study if they had any risk of health damage when taking part in an MRI scan (e.g., were pregnant, had metal on/in their body, or a pacemaker). Further exclusion criteria were the presence or the suspicion of a neurological disorder, current or previous addiction to or heavy abuse of any substance based on the Structured Clinical Interview for the Diagnostic Schedule for Mental Disorders–Fourth Edition (SCID-I and -II; [41]), use of any benzodiazepine on the day of data collection, or a lifetime diagnosis of schizophrenia or bipolar disorder, because these can cause neurochemical alterations that could possibly confound the investigation. Participants could only be included as HCs if they did not have a history of psychiatric diagnosis and did not use centrally active medication. Accordingly, twenty HCs (mean age (SD) = 24.90 (6.28) were approximately matched with twenty participants with diagnosed BPD (mean age (SD) = 24.85 (5.16), t = −0.027, *p* = 0.978). The sample sizes are consistent with recommendations for statistically reliable results in fMRI studies [42]. Both groups did not differ significantly in their intelligence quotient measured by the language-based intelligence test [40] (mean HC (SD) = 116.80 (6.71), mean BPD (SD) = 110.80 (14.10), t = −1.178, *p* = 0.094). However, there was a difference with regard to the level of education, as all HCs had earned a high-school diploma but not all BPD patients had (χ^2^ = 7.059, *p* = 0.029), which is a known fact about BPD, that those affected remain below their means in terms of education. The visual functioning of each participant was tested before starting the fMRI measurement.

### 2.2. Measures

Diagnoses were assessed with the SCID-I and SCID-II [41]. The differentiation of the groups into BPD patients and HCs was additionally ensured with the Borderline Personality Inventory (BPI, [43]) a self-report questionnaire for BPD symptomatology. The internal consistency of the measure was very good (α = 0.94 [0.92, 0.97]) and BPD patients scored significantly higher than the HCs (t = 10.52, df = 28.81, *p* < 0.001). We used the Heidelberger Fragebogen zu Schamgefühlen (HFS; [44]), a self-report questionnaire, to assess the sensitivity for shame with regard to “body and sexuality” and “performance and social competence”. Participants were asked to rate the intensity of shame that a person in a described scenario feels on a Likert scale from 1 (not at all) to 6 (very strong). The internal consistencies of the scales were α = 0.86 [0.79, 0.92] and α = 0.91 [0.86, 0.95], respectively. The overall scale of the German version of the Interpersonal Guilt Questionnaire (IGQ; [45]) was used to measure guilt. Patients rated their agreement with items regarding guilt in relation to survival, separation, and responsibility on Likert scales ranging from 1 to 5 (α = 0.89 [0.83, 0.93]).

### 2.3. Magnetic Resonance

To collect fMRI images, we used a Philips 3-Tesla scanner (Philips Achieva 3.0 T TX; Philips Medical Systems; Best, The Netherlands) with a 32-channel head coil. For anatomical reference, T1-weighted scans (220 slices of 1 mm thickness) were obtained [time of repetition (TR): 8.2 ms, time of echo (TE): 3.7 ms, flip angel (FA): 8°, Matrix: 240 × 187, field of view (FOV): 188 mm × 240 mm × 220 mm, voxel size: 1 mm × 1 mm × 1 mm, sagittal slices]. For the functional data, Blood Oxygen Level Dependency (BOLD) contrasts were acquired with T2*-weighted echo-planar imaging sequences. 36 axial slices (standard AC-PC orientation) with a thickness of 3 mm were scanned [TR: 2000 ms, TE: 30 ms, FA: 90°, Matrix: 76 × 79, FOV: 224 mm × 236 mm × 108 mm, voxel size: 3 mm × 3 mm × 3 mm]. In each of the two runs of the experiment, 316 whole-brain volumes were obtained.

### 2.4. Experimental Stimuli

**Figure 1:**
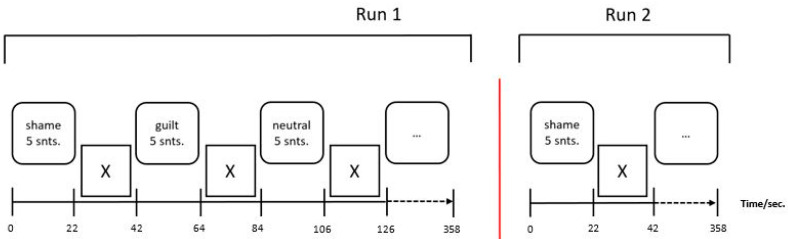
Sequence of the experiment in fMRI environment.

The experimental paradigm was presented in the MRI scanner via a head-coil compatible mirror system (300 cm screen to mirror, 15 cm mirror to participant’s eyes). Verbal written stimuli were projected on a translucent screen in white capitals (font: Arial, font size: 16) on a black background by a commercially available video beamer (INTouch, resolution of 1024 × 768 pixel) protected by a Faraday cage. Presentation and timing were implemented using the software Presentation^®^ (version 14.9, Neurobehavioral Systems). The paradigm used in this study is part of a larger research project [46] and has been developed and validated in previous studies [18,30]. Those studies showed the paradigm to be able to induce the intended emotions. Participants were asked to imagine situations that were described to them in sentences (Appendix A). In this study we used sentences evoking shame (e. g. “I’m sweating under my arms.”) or guilt (e.g., “I knock over an old lady.”) or neutral sentences (e.g., “I’m brushing my teeth.”). The neutral condition was intended to be the experimental baseline, which the shame and guilt condition would be contrasted against. Over the course of the experiment, the patients saw 15 sentences for each condition written from a first-person perspective. A block design was used, in which a block consisted of 5 sentences of the same condition (shame, guilt, or neutral). Each sentence was presented for four seconds (enough time for one scan) with a 400 ms pause between sentences, to account for a time-lagged BOLD signal. There was a 20 s break between blocks (enough time for five scans), to normalize the cortical activity before continuing with the next experimental condition. Participants confirmed reading and understanding each sentence by pressing a button. Nine blocks of sentences were presented in one *run*. Two runs were performed to increase measurement accuracy. The experiment and scanning procedure lasted a total of 30 min. Figure 1 shows a schematic diagram of the experiment.

Half an hour after the scanning procedure, participants were asked to rate the degree to which each sentence presented during the experiment elicited feelings of shame and guilt and to what degree the sentences were neutral to them, on a Likert scale ranging from 0 to 4.

### 2.5. Statistical Analysis

Analyses were carried out using SPSS 25 [47] and R [48]. Data from the questionnaires and interviews were compared between groups using *t*-tests and χ^2^-tests. Associations between measures were presented using Pearson’s r. All analyses were two-tailed and a significance threshold of *α* = 0.05 was applied.

To analyze the fMRI data, we used SPM8 [49] which is embedded in MATLAB R2012a [50]. Pre-processing included slice scan time correction, functional-to-anatomical co-registration based on the Montreal Neurological Institute (MNI) template, spatial normalization using segmentation, and temporal smoothing. Motion correction was performed using the first functional image of the brain in the scan as a reference. Correction was performed in three translational and three rotational directions, as the position of the head can shift during the measurement in six different axes. The extracted motion regressors were later used as factors in the model specification. Afterwards, the BOLD signal in each voxel was predicted, first (1st level, person) through a fixed effects model with the predictors emotion condition (neutral, shame, and guilt) as well as rotation and translation parameters derived from preprocessing, and second (2nd level, group) through a flexible factorial model with subject (flexible factor derived from the 1st-level model), group (BPD or Control) and condition (neutral, shame, guilt). On a person-level, the significance threshold for contrasts was set to α = 0.001 (uncorrected). Because of the small sample, contrasts on the group level were examined with a significance threshold of α = 0.05 (uncorrected) with a minimal cluster size of five voxels. Voxel-based contrast maps were computed for each condition. The anatomic coordinates of voxel groups that displayed significant contrasts were localized on a standard stereotactic atlas [51] using TalairachClient (Version 2.4.2, http://www.talairach.org, accessed on 24 January 2013).

Descriptive statistics and *t*-tests were used to examine between-group differences on the HFS and IGQ. Similarly, within-group differences in the appraisal of experimental stimuli were examined. For example, the mean shame rating for the shame sentences was compared to the guilt and neutral rating in pairwise *t*-tests.

## 3. Results

### 3.1. Self-Reported Disposition

Between-group differences regarding self-reported dispositions to feel shame and guilt are described in Table 1. In contrast to our expectations, the differences in self-reported disposition to feel shame between BPD patients and the HCs were not significant. However, BPD patients reported more interpersonal feelings of guilt than the HCs.

### 3.2. Experimental Stimulation

The BPD patients and HCs did not differ in the reported feeling of shame when reading the shame sentences (*M_pat_* = 2.54, *M_con_* = 2.58, *t*(38) = −0.17, *p* = 0.866) or of guilt when reading the guilt sentences (*M_pat_* = 2.34, *M_con_* = 2.60, *t*(38) = −1.07, *p* = 0.293). Significant intra-group differences in the shame, guilt, and neutral appraisal regarding all experimental conditions appeared in both groups (all *p* < 0.001), supporting the success of the experimental stimulation.

### 3.3. fMRI: Contrast Analysis

Contrasting the neutral condition of the experiment with any other of the experimental conditions elicited numerous large-scale activation clusters in the fMRI scanner.

**Figure 2:**
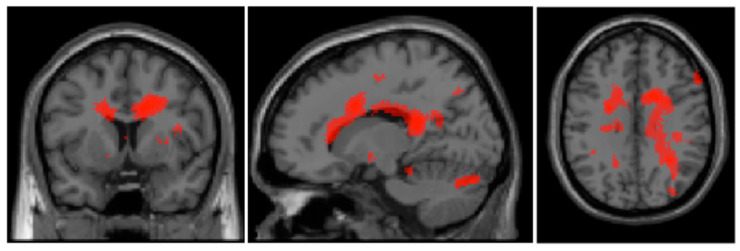
T-contrast activations “shame_pat” > “shame_con”: right cingulate gyrus; right cerebellum, declive, pyramidal; left gyrus fusiformis (Peak-level: *p* < 0.05, uncorrected; statistical pictures showing the activation on a color scale from dark to light red with darker shades symbolizing stronger activation and lighter shades symbolizing weaker activation).

Stark and Squire [52] showed how such clusters can impede the integrity of contrasts analyses, which is why we did not use the neutral condition as a baseline and, in the following, only report the between-group contrast of the experimental conditions. The between-group contrast (BPD patients vs. healthy controls) regarding the shame condition revealed two significant clusters. The first one was located primarily in the limbic regions of the right hemisphere (the Brodmann region 23/24/31/33). The second cluster included in the right hemispherical the following regions: posterior (cerebellum, declive, pyramidal) and occipital (fusiform gyrus, Brodmann region 19) areas. This contrast is visualized in Figure 2. For the reverse contrast *(healthy controls vs. BPD patients)* a significant cluster emerged in the left hemispherical in the occipital regions (Brodmann-region 18), specifically including the cuneus and gyrus lingualis. Further details on the clusters are listed in Table 2.

**Figure 3:**
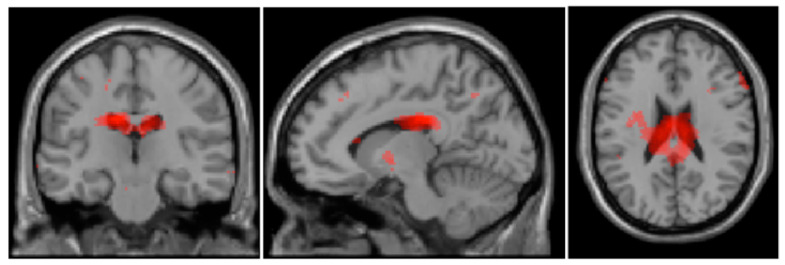
T-contrast activations “guilt_pat” > “guilt_con”: left and right cingulate gyrus, left caudate nucleus; right cerebellum, declive, pyramidal, right gyrus fusiformis; left gyrus fusiformis, gyrus occipitalis inferior, cerebellum, declive (peak level: *p* < 0.05, uncorrected; statistical pictures showing the activation on a color scale from dark to light red with darker shades symbolizing stronger activation and lighter shades symbolizing weaker activation).

For the between-group contrast in the guilt condition, one large cluster with peaks at the nucleus caudatus and in the limbic regions (gyrus cinguli, Brodmann region 23) emerged as significant. Two smaller clusters were found around the gyrus fusiformis and gyrus occipitalis inferior (Brodmann region 19), and in the posterior regions (cerebellum, pyramis, declive). This contrast is visualized in Figure 3. In contrast to the BPD patients, stronger activations were detected in the cuneus, gyrus lingualis, gyrus praecentralis, gyrus postcentralis, gyrus temporalis superior, and uncus of the HCs. Further details on the clusters can be seen in Table 3.

## 4. Discussion

The present study investigated the experience of shame and guilt in patients with BPD and their neural processing. We expected BPD patients to report higher levels of dispositional (general) and situational shame and guilt induced by experimental stimulation than the HCs. In accordance with this, we expected neural activation in BPD patients to be stronger in the known regions related to shame and guilt processing, namely the insula, cingulate cortex, TPJ, dlPFC, as well as in the sensor- and premotor cortex [32], compared to the HCs.

### 4.1. Self-Reported Disposition to Shame and Guilt in BPD

In our study, BPD patients reported higher levels of interpersonal guilt than the HCs. In line with our findings, Rüsch and colleagues [8] as well as Spitzer and colleagues [4] documented increased (implicit and explicit) reports of guilt in BPD patients, emphasizing the importance of this emotion in BPD. However, in our study, no significant between-group-differences were found for shame regarding body and sexuality or performance and social competence. Differences in findings of self-reported guilt and shame might be due to different measures, also in our study it might be due to the shame questionnaire, the HFS, used in this study. The German questionnaire, the HFS, asks participants to rate the degree to which a person in a scenario is feeling ashamed, but not to what degree they themselves would be ashamed. This questionnaire lists generally applicable scenarios but no self-related scenarios. However, in BPD, emotional reactivity seems to be specific to individually self-related important contexts [53]. In a meta-analysis by Buchman-Wildbaum et al. [2], the presence of a BPD diagnosis had large effects on the reported levels of shame but based on measures other than the HFS. In a recent study by Biermann et al. [24], BPD patients reported higher levels of shame-proneness (measured with the Test of Self-Conscious Affect; TOSCA-3, German version; [54]) compared to HCs. However, experimental manipulation of the focus towards oneself vs. others leads to more reported shame in HCs compared to more reported self-disgust in BPD patients. This indicates that the degree to which a scenario presented in a questionnaire relates to the feeling of shame varies with the degree to which the reader can attribute the scenario to him-/herself. Further, this mechanism seems to vary between BPD patients and HCs.

### 4.2. Experimental Stimulation

All participants reported heightened feelings of shame and guilt during the respective sentences. This accords to previous data supporting the potency of the experimental paradigm [18,30]. However, BPD patients did not report stronger feelings of shame or guilt than healthy controls after reading these sentences. The sentences were validated in a healthy sample and do not refer to a BPD-specific context. This might be the reason why the rating of emotionality of the sentences did not differ between patients and HCs. However, even though the emotional intensity did not differ, differences in neural processing might be informative with regard to different cognitive processes that may be involved in shame and guilt in BPD patients and HCs [23,24]. One reason for comparable judgements in BPD and healthy participants might be that patients with BPD know about the most common, socially frequent judgements of healthy others. Since the sentences are more distanced when being judged than during experimentally induced imagination this could be one effect of situational differences with regard to the stimulation of the emotional response. However, another reason might be the assumed effort to regulate the affective response in BPD participants reflected by activation of control-related brain networks during the imagination of both social emotions instead of focusing on emotional imagination and experiencing when being compared to healthy participants.

Consequently, one might assume that although participants with BPD can successfully regulate their affective experience of shame and guilt, this is associated with higher neurofunctional effort compared to healthy participants.

While evidence suggests that individuals with BPD may not have difficulties implementing different emotion regulation strategies [55,56,57], it is possible that this patient group does have to exert much more effort to implement the strategies that are best suited to a given level of arousal during the experimental task. Another explanation might be that in patients that are diagnosed with BPD emotional cues are only emotionally vulnerable when borderline-specific schemes are triggered, e.g., concrete aversive or ambiguous interpersonally relevant cues [58]. Finally, it has been shown that the conscious application of emotion acceptance reduced insular activation in individuals with BPD [59]. Probably, our shame and guilt scenarios are known to be acceptable, since the scenarios are not BPD-specific and could happen to anyone. This corresponds with difficulties in identifying and being aware of emotional experiences after imagination of the social scenarios. This may support the assumption that BPD patients may use cognitive routines such as emotion suppression but have to exert more effort to regulate though reporting the same experiencing of these emotions.

### 4.3. Functional Imaging

Despite the lack of differences in the self-report of experimentally induced shame and guilt, meaningful differences during the neural processing of these emotions were detected. During the shame condition, stronger activations in the posterior and anterior cingulate cortex were found in BPD patients compared to controls. The cingulate cortex is often credited with self-referential processing or cognitive emotional regulation and control of negative stimuli/harmful and stressful experiencing or morally negative emotions [32,55,60,61,62]. Additionally, stronger activations for BPD patients than for healthy controls were found in the gyrus fusiformis, which has been discussed in the literature previously in association with emotional processing of negative stimuli in BPD patients [63,64]. This might be interpreted as increased attempts of BPD patients to regulate emotions, especially by cognitive means, possibly leading to problems in detaching themselves from these emotions. As part of the pronounced cognitive processes, BPD patients seem to need more effort to regulate self-relevance in socio-emotional situations related to shame and guilt than HCs, e.g., by self-evaluating in comparison to others. Van Schie et al. [36] and Orth et al. [37] both found this process to be altered in BPD patients compared to HCs.

Compared to BPD patients, healthy participants showed increased activation in the cuneus and the gyrus lingualis, which are thought to play a role in the induction of emotion through visually presented stimuli [65] and imaginative experience [66,67]. Possibly, this represents a stronger tendency of HCs to actually imagine the induced socially and emotionally relevant situation and engage with that individually created image, compared to BPD patients. That could indicate that shame related processes might be more harmful and self-attacking in the patient group, thus experiencing shame has to be avoided.

In the guilt condition, BPD patients showed significantly stronger activations than HCs in regions associated with emotion-regulative processing. Comparable to the shame condition, stronger activations in the posterior cingulate cortex and the gyrus fusiformis are linked to stronger self-referential processing of negative social stimuli [64]. Stronger activations for BPD patients than for HCs in the nucleus caudatus may reflect an increased focus to plan and execute actions [68,69]. Since guilt, as opposed to shame, is less connected to a negative evaluation of the whole self but of a specific self-driven action [70], it is plausible that BPD patients think about different ways of acting when feeling guilty as emotionally regulative behavior. Again, during the guilt condition, the HCs showed stronger activations compared to the BPD patients in the cuneus and gyrus lingualis, probably indicating stronger imagination of the stimulus material. Additionally, since the HCs showed stronger activations related to the hippocampus and the limbic uncus, a more memory-pronounced processing of the stimuli may be assumed [71,72]. These findings might therefore indicate that HCs are more willing than BPD patients to imagine and engage with scenarios of socio-emotional relevance, while emotion regulation processes are less pronounced, because the scenarios might be less threatening for the HCs. In line with the results of Michl et al. [30], the HCs in our study showed stronger activations compared to the BPD patients within a fronto-temporal network. Activations in the frontal lobe may reflect the application of social norms or of regulative standards, while activations in the temporal lobe might be associated with conclusions about the thoughts and intentions of others [73,74].

Different patterns of pronounced cortical activation during the processing of shame and guilt were identified for BPD patients and healthy controls. The regions that emerged from this contrast analysis have—in part—been named important in the processing of guilt and shame in general [31,32]. However, we conclude that different processes are distinctly pronounced in each group: the activation patterns shown by the HCs indicate increased memory-related processing, stronger imagination processes, and a stronger focus on social relevance compared to the BPD patients. We interpret this as HCs being more open to and feeling less threatened by engaging in the social and self-relevant emotions of shame and guilt, which would be in line with a tendency of BPD patients to avoid exposure to aversive emotional, self-relevant scenarios, and memories. Meanwhile, the activation patterns presented by BPD patients speak to increased self-referential processes regulating the experience by cognitive means. This could indicate an increased awareness for the socio-emotional cues for shame and guilt, which could mean further threats, as well as pronounced attempts to regulate the emotional experience, and a greater difficulty for patients to detach themselves from shame and guilt. Our findings are largely in accordance with the results by Orth et al. [37] and van Schie et al. [36]. Particularly, the increased activation in the cingulate regions underscores the notion of a pronounced emotional and self-referential processing by BPD patients compared to controls. This is in line with findings in other psychiatric patients [18,33].

### 4.4. Limitations

On the behavioral level, limitations include the partial failure to differentiate between BPD patients and healthy controls in self-reported guilt and shame, especially during the experiment. The paradigm was developed for and validated in a sample of healthy individuals [30,74], to be generally applicable. It also revealed meaningful differences between the healthy and pathological groups [18]. However, it possibly lacks specificity to BPD-specific interpersonal and trauma-related problems [75]. Future studies might enhance the given paradigm by using trauma-related and disorder-specific or even individualized shame-/guilt-inducing stimuli [76]. On the brain activation level, we found, in contrast to previous applications of the experimental paradigm, numerous large activation clusters when comparing the neutral condition to the shame or guilt condition. A reason for the large activations revealed by this contrast might be that the neutral sentences described actions that patients might have been able to strongly visualize and imagine. Another reason could be the high autobiographical reference of some sentences that might be even more threatening for patients with BPD. This restricted the use of the neutral condition as a baseline comparison [52] and hindered our analysis in isolating the neural processing of both emotions from general neural activity and limited the analysis to group comparisons. Thus, Hypothesis 3 could not be tested. Even though the sample size corresponded to recommendations in the literature, small sample sizes are associated with reduced statistical power [77], which is particularly problematic for the analysis of neurofunctional data. Hence, our analysis would not have been able to detect smaller effects. Also, as the two groups differed regarding their educational level, future studies should examine the influence of education on the neural processing of guilt and shame.

## 5. Conclusions

The present study does not only replicate findings of enhanced guilt-proneness in BPD patients and produces results supporting the importance of the cingulate cortex in processing social emotions but also demonstrates the divergence between subjective self-reports and neurobiological processing. The differences regarding self-report measures in our sample were scarce and thus insufficient to support Hypothesis 1 and 2. However, based on neural activation, we found evidence for important differences in the processes behind the experience of shame and guilt during an imagination task between BPD patients and HCs, supporting Hypothesis 4.

Neural activations during the experiment on the one hand indicate that HCs tended to engage more in social emotions than BPD patients via the imagination, autobiographical integration, and classification of the significance of shame- and guilt-related sentences.

The neural activations of the BPD patients, on the other hand, indicated a stronger perception-related activation, increased effort in cognitive emotion regulation, as well as increased self-referential processes compared to the HCs.

Hence, it can be concluded that BPD patients have to focus their cognitive and general regulative resources during conflicting social situations to perform like healthy persons and avoid affective threatening experiencing. Probably, this mechanism prevents situational increasing arousal, tension, and dissociation, but results in emotional and mental perception as well as pain avoidance [78].

Our results suggest that treatments for BPD should support patients to engage with social emotions in a different way. Shifting the focus away from the own person and from self-perception but towards the emotional situation, which might—among other things—be supported through imagination exercises, as it has recently been shown that acceptance-based and validation-based approaches are superior to other regulative approaches, e.g., like cognitive suppression [79,80] and even reframing [81].

## Figures and Tables

**Table 1:** Between group differences in the disposition to feel guilt and shame.

Measure	Scale	Mean (SD) or % BPD Patients	Mean (SD) or % Healthy Controls	*t*(df)	*p*
HFS	Body and sexuality	76.53 (14.55)	70.15 (12.55)	1.47 (37)	0.151
	Performance and social competence	71.75 (17.16)	67.65 (17.34)	0.75 (38)	0.457
IGQ	Total	65.80 (11.91)	52.65 (13.50)	3.27 (38)	0.002

Note: BPD = borderline personality disorder; HFS = Heidelberger Fragebogen zu Schamgefühlen (Heidelberg Shame Questionnaire); IGQ = Interpersonal Guilt Questionnaire.

**Table 2:** Comparison of neural activation during the shame condition between BPD patients and healthy controls (between-group comparison).

Cluster	Cluster-Size	Brain Regions	Hemisphere	BA	MNI-Coordinates (x, y, z)			*T*
Shame: BPD patients > healthy controls								
1	6266	Gyrus cinguli	R	24	16	8	32	4.12
		Cingulum posterior	R	23	14	−38	18	4.07
		Cingulum anterior	R	33	10	26	12	3.58
2	1273	Declive (Cerebellum)	L	*	−30	−82	−16	3.56
		Gyrus fusiformis	R	19	32	−80	−10	3.46
		Pyramis (Declive)	R	*	16	−80	−26	2.98
Shame: healthy controls > BPD patients								
1	1920	Cuneus	L	18	−2	−94	12	3.22
		Cuneus	L	18	−8	−88	32	2.70
		Gyrus lingualis	L	*	−14	−74	6	2.63

Note. R = right, L = left, BA = Brodmann region; *T* = t-value; significance threshold peak level: *p* < 0.05 (uncorrected). *: Brodmann areas are exclusively a categorisation for areas of the cerebral cortex, the Cerebellum isn’t included.

**Table 3:** Comparison of neural activation during the guilt condition between BPD patients and healthy controls.

Cluster	Cluster-Size	Brain Regions	Hemisphere	BA	MNI-Coordinates (x, y, z)			*T*
Guilt: BPD patients > healthy controls								
1	2302	Nucleus caudatus	L	*	−12	−22	28	6.22
		Gyrus cinguli	L	23	−4	−12	24	4.62
		Gyrus cinguli	R	23	12	−16	26	4.16
2	890	Gyrus fusiformis	R	19	32	−80	−10	4.38
		Pyramis (CB)	R	*	14	−80	−26	2.92
		Declive (CB)	R	*	6	−82	−20	2.37
3	872	Declive (CB)	L	*	−28	−82	−14	3.76
		Gyrus occipitalis inferior	L	19	−44	−82	2	3.62
		Gyrus fusiformis	L	19	−46	−76	−8	2.78
Guilt: Healthy controls > BPD patients								
1	7154	Cuneus	R	18	6	−78	24	4.05
		Gyrus lingualis	L	*	−14	−74	6	4.02
		Gyrus lingualis	R	18	16	−68	2	4.00
2	1279	Gyrus praecentralis	R	6	62	2	22	3.73
		Gyrus praecentralis	R	4	52	−12	44	3.00
		Gyrus postcentralis	R	4	62	−10	32	2.86
3	838	Gyrus temporalis superior	R	22	64	14	−8	4.03
		Gyrus temporalis superior	R	38	62	14	−16	3.40
		Uncus	R	36	24	−4	−34	3.24

Note. R = right, L = left, BA = Brodmann region, CB= cerebellum; *T* = t-value; significance threshold peak level: *p* < 0.05 (uncorrected).*: Brodmann areas are exclusively a categorisation for areas of the cerebral cortex, the cerebellum isn’t included.

## Data Availability

The data can be made available upon request by the corresponding author.

## Appendix A

**Table A1:** Original German experimental sentences and the translated English sentences.

Deutsch	English
Scham	Shame
Ich muss im Krankenhaus eine Bettpfanne benutzen.	I have to use a bedpan in hospital.
Ich werde beim Nase bohren beobachtet.	I am being watched while picking my nose.
Ich habe Mundgeruch.	I have bad breath.
Ich pupse laut in der Kirche.	I fart loudly in church.
Ich verwechsle einen Fremden mit meinem Partner.	I mistake a stranger for my partner.
Ich rülpse laut im Restaurant.	I burp loudly in a restaurant.
Ich spucke beim Sprechen.	I spit when I speak.
Ich treffe meinen Chef in der Sauna.	I bump into my boss in the sauna.
Ich schnarche laut während eines Vortrags.	I snore loudly during a lecture.
Ich werde von Nachbarn beim Sex beobachtet.	I am watched by neighbors during sex.
Ich bemerke, dass meine Hose offen steht.	I notice that my pants are open.
Ich bin der einzig verkleidete auf einer Party.	I am the only person dressed up at a party.
Ich erkenne einen alten Bekannten nicht.	I don’t recognize an old acquaintance.
Ich schwitze sichtlich unter den Armen.	I’m visibly sweating under my arms.
Ich pinkele in die Hose.	I pee in my pants.
Schuld	Guilt
Ich teile mein Wasser nicht mit jemand durstigem.	I don’t share my water with someone thirsty.
Ich habe kaum Zeit für meinen Partner.	I barely have time for my partner.
Ich schicke einen Touristen in die falsche Richtung.	I send a tourist in the wrong direction.
Ich hindere jemanden nicht, Fehler zu begehen.	I don’t stop someone from making mistakes.
Ich schlage einem Freund einen Gefallen aus.	I refuse to do a friend a favor.
Ich überfahre einen Igel.	I run over a hedgehog.
Ich übertrage eine Grippe an Kollegen.	I pass on the flu to colleagues.
Ich ruhe mich auf Lorbeeren anderer aus.	I rest on the laurels of others.
Ich mache Urlaub, während mein Partner im Krankenhaus liegt.	I go on vacation while my partner is in the hospital.
Ich mobbe Kollegen.	I bully colleagues.
Ich remple eine alte Dame um.	I knock over an old lady.
Ich war lange nicht mehr am Grab meiner Großeltern.	I haven’t been to my grandparents’ grave for a long time.
Ich sage kurzfristig ein wichtiges Gespräch ab.	I cancel an important meeting at short notice.
Ich nehme keine Rücksicht auf andere.	I show no consideration for others.
Ich stolpere über einen Welpen.	I trip over a puppy.
Neutral	Neutral
Ich falte eine Decke und lege diese auf das Sofa.	I fold a blanket and lay it on the sofa.
Ich knipse das Licht an.	I switch on the light.
Ich rühre die Milch im Kaffee um.	I stir the milk in my coffee.
Ich nehme ein Stück Seife um meine Hände zu waschen.	I take a bar of soap to wash my hands.
Ich wische den Tisch ab.	I wipe the table.
Ich spaziere gemütlich zum Bus.	I take a leisurely stroll to the bus.
Ich putze mir die Zähne.	I brush my teeth.
Ich creme mir die Hände ein.	I put lotion on my hands.
Ich schüttele die Kissen meines Bettes auf.	I fluff the pillows on my bed.
Ich blättere in einem Katalog.	I leaf through a catalog.
Ich gieße die Blumen auf meinem Balkon.	I water the flowers on my balcony.
Ich esse zu Abend.	I eat dinner.
Ich verschließe einen Brief.	I seal a letter.
Ich reiße eine Milchtüte auf.	I tear open a carton of milk.
Ich trinke in meiner Pause einen Kaffee.	I drink a coffee during my break.
